# Why incorporating benazepril into a renal diet may improve treatment efficiency and compliance in cats with chronic kidney disease

**DOI:** 10.3389/fvets.2026.1872196

**Published:** 2026-09-16

**Authors:** Alice Bidaud, Jennifer Tardiveau, Alice Couronné, Terence Pellet, Agnès Batard, Laurent Frayssinet

**Affiliations:** Virbac SA, Carros, France

**Keywords:** benazepril, cat, chronic kidney disease, compliance, medicated diet, treatment efficiency

## Abstract

Treating cats with chronic kidney disease generally requires daily administration of an antiproteinuric drug, which can impact medication compliance with medical outcomes as well as negative implications for the wellbeing of the cat and the owner. In addition, for a given daily dose, benazepril is more effective when administered several times a day but this could increase non compliance. It was therefore conceived to incorporate benazepril into a highly palatable diet for cats with chronic kidney disease, that is, a medicated diet, that the cats could consume throughout the day, with the aim of replacing multiple administrations thanks to the cat's nibbler eating behavior and the slow gastric emptying in cats eating dry food. Our investigation showed that Consumption of the medicated diet led to an overall lower drug exposure in both healthy and CKD cats compared to benazepril once-daily tablets. Nevertheless, the overall efficacy in inhibiting ACE was at least equivalent (CKD cats) and even higher (healthy cats). In both cases, this resulted in greater efficiency (i.e., the amount of desired therapeutic effect produced per unit of drug exposure) of the medicated diet than of conventional once daily tablet administration. More broadly, this observation suggests that the temporal pattern of benazepril exposure may be more important than the total dose administered. Medicated diets may represent a new strategy for improving both compliance and therapeutic efficiency in chronic feline diseases, provided that the diet is well accepted by the cat.

## Introduction

Chronic kidney disease (CKD) is a very common disease in older cats, with a prevalence of up to 60% in cats older than 10 years and even 80% in cats over 15 years old (1).

Proteinuria, defined as Urine protein to creatinine ratio higher than 0.4, is observed in 20%−30% of CKD cats (2–4) and is known to promote disease progression (5, 6) and impact life expectancy (7–9). Therefore the management of CKD cats with proteinuria requires daily treatment with an anti-proteinuric drug (1, 10). Benazepril is a widely used antiproteinuric drug and is currently available in the form of tablets to administer daily.

Unfortunately, cat owners face several challenges when medicating chronically their cats, often leading to uncompleted courses of medication and even treatment cessation. Medication non-compliance is reported in nearly 40% of cat owners. For many of them, administering medication to their pet is challenging, the most frequently reported challenge being the cat spitting out the tablet (11, 12). Clients' inability to medicate their cats may have implications for clinical outcomes and may damage the relationship between owners and their pets and even increase the risk of “caregiver burden” (11, 13). In this context, alternatives to facilitate drug administration are desirable (12, 14).

On the other hand, efficacy for a given dose unit, that is efficiency, has been shown to be improved when benazepril is administered twice daily or more (15–17). Based on simulations, it has been shown that the ideal plasma exposure profile to increase the efficiency of benazeprilat is that of continuous infusion. More recently, Schneider et al. demonstrated that splitting the daily dose of benazepril into two administrations (q12h) rather than a single daily dose (q24h) resulted in more robust downregulation of classical RAAS biomarkers and upregulation of alternative RAAS biomarkers compared to a single daily dose. Additionally, q12h dosing led to fewer fluctuations in angiotensin levels in plasma compared to q24h dosing, which may contribute to more consistent therapeutic effects (17). However obtaining an ideal plasma profile through repeated daily administrations comes up against increased risks of non-compliance.

Since cats are grazers that consume 8–16 meals per day, incorporating benazepril into a renal support diet, a cornerstone of the management of CKD cats (2) regardless of the proteinuria, would allow for the replacement of multiple daily administrations of benazepril, without any additional burden for the owner other than feeding their cat the recommended ration of this medicated diet.

The objective of this study conducted in both laboratory and field conditions was to explore the efficacy of the medicated diet distributed once a day and left available to the cat for 24 h compared to conventional tablets given once daily.

## Materials and methods

The in-life phase of the study was conducted in two parts: part I in healthy cats in laboratory conditions (divided into parts Ia and Ib) and part II in CKD cats in field conditions.

### Animals

Twelve healthy European adult male cats weighing between 3.67 and 4.48 kg were involved in part Ia and 12 healthy European adult female cats, weighing between 2.5 kg and 4.15 kg in part Ib. They were housed individually in pens on welfare grounds, in a climate controlled environment and in compliance with current regulations, for the duration of the trials. The animals were fed once a day in the morning (either the medicated diet or the blank renal diet). The food was left available until the next distribution (about 24 h) and tap water was available *ad libitum*.

In Part II, 106 CKD cats of any breed, age, status (i.e., intact or neutered) and gender, were included from client households in 26 veterinary clinics from France, Germany, Hungary and Portugal. 61% of the cats were at IRIS stage 2, 23% at stage 1 and 16% at stage 3. All had been treated with benazepril tablets for at least 1 month prior to their inclusion because they had been diagnosed with proteinuria. The mean age at enrolment was similar with 129.7 and 129.8 months (i.e., 10.8 years) in the medicated diet group (MD group) and the control group, respectively. The same applied for mean body weight with 4.1 and 4.2 kg BW for the MD group and the control group, respectively. The cats were kept with their owners under their usual housing conditions.

This research received ethical approval from Klifovet Animal Welfare Body (Register No. 18-05) in January 2018. Written informed consent has been granted by the catowners.

### Blank renal diet and medicated diet compositions

The blank renal diet was specially formulated in accordance with European regulation to support renal function in case of chronic renal insufficiency by reducing protein and phosphorus levels (in g/Mcal; protein 72, fat 50, phosphorus 1.08, calcium 2.2, ω3 fatty acids 2.64, EPA+DHA 0.96). To compensate for the common decrease in appetite seen in CKD cats, the blank renal diet was rich in calories (Metabolisable energy 4.16 Mcal/kg). The medicated diet had the same nutritional formula as the blank renal diet, to which had been added the amount of benazepril necessary to provide a minimal dose of 0.5 mg/kg/day to the cat, assuming the complete consumption of the daily ration. Efforts have been made to make the diet highly palatable and subsequent palatability tests and field feedback indicated very good palatability.

### Study design

In Part Ia and Ib, a parallel design was used with 6 cats per group (Control group and MD group). So in total, 12 healthy cats were assigned to the Control group and 12 other healthy cats to the MD group.The Control group was administered Fortekor^®^ 2.5 mg tablets for cats and dogs and the MD group was fed the medicated diet with benazepril. In the Control group, benazepril was orally administered at the theoretical dose range of the reference product Fortekor^®^, that is, 0.5 to 1 mg/kg body weight once daily for 8 days. Animals of the Control group were fed the blank renal diet just after administration of Fortekor^®^ tablets. The MD group received 0.5–1 mg benazepril/kg/day through the medicated diet assuming complete consumption of the prescribed ration. In the MD group, the consumption was evaluated at 0.5, 2, and 6 h post-feeding and before next feeding. Blood samples were regularly collected over the 8 days of treatment. In part Ia, blood samples were taken before each daily treatment, and at 2, 6, 8, and 12 h post treatment on days 1, 4 and 8, plus 24 h and 48 h post treatment on the 8th day. In part Ib, blood samples were taken before treatment on days 1, 2, 4, 6, and 8, and at 2, 4, 6, 8, and 12 h post treatment on days 1 and 8, plus 0.5, 16, 24, and 48 h post treatment on the 8th day.

In part II, after a dietary transition with the blank renal diet, 38 cats were allocated to the Control group and received Fortekor^®^ tablets (0.5–1 mg benazepril hydrochloride/kg/day) with the blank renal diet, while 74 cats were allocated to the MD group and received the medicated diet (0.5–1 mg benazepril hydrochloride/kg/day assuming complete consumption of the prescribed ration). Animals were randomized in the sequence of enrollment on D0 in a ratio MD group: Control group of 2:1 to maximize the collection of data for the new formulation while keeping the reference control group to an optimized, ethically appropriate size. The study duration was 6 months. Blood and urine samples were taken on day 0 (D0), day 30 (D30), day 90 (D90) and day 180 (D180) for each cat.

### Determination of plasma benazeprilat concentrations

Benazeprilat was assayed in cat plasma using validated LC-MS/MS methods. The range of quantification was between 0.5 ng/mL (LLOQ) and 100 ng/mL (ULOQ).

### Determination of serum angiotensin converting enzyme (ACE) activity

In Part Ib and Part II, ACE activity was measured in cat serum with a colorimetric kit method PKH/MOA/1153. The limit of quantification of the method was 12 U/L for undiluted samples. As all samples needed to be diluted by half to prevent any matrix effect, the applied LLOQ was 24 U/L.

### POP PK/PD analysis

Population pharmacokinetic and PK/PD analyses were performed using nonlinear mixed-effects modeling (NLMEM) Data analysis (Phoenix^®^WinNonlin^®^8.3.5, Certara, Princeton, New Jersey, USA), that is the appropriate tool for the meta-analysis of unbalanced data sets (*n* = 12 cats for Part Ia, *n* = 12 for Part Ib and *n* = 106 for Part II). The base benazeprilat structural model was of the class of physiologically-based models developed for benazeprilat in dogs by Toutain et al. (15) and subsequently used for Fortekor^®^ in cats (16). It incorporated interindividual variability, residual unexplained variability, and covariate effects. Parameters estimation was performed using the maximum likelihood method, with appropriate handling of observations below the limit of quantification (M3 method). Model evaluation included standard goodness-of-fit diagnostics, assessment of parameter precision by bootstrap resampling, evaluation of random-effect shrinkage, and visual predictive checks based on simulated datasets. These complementary procedures demonstrated that the model adequately described both the central tendency and variability of the observed PK and PK/PD data [see (18) for a review or (17) for a more recent view of this question].

The difference between the two formulations was evaluated based on the exposure to the active fraction of benazeprilat (AUCfree: area under the concentration–time curve of the free fraction of benazeprilat), the total exposure to benazeprilat [AUCtot: area under the concentration–time curve of the measured (total) plasma concentration], the efficacy regarding ACE inhibition (AUEC: area under the ACE activity-time curve) and the pharmacodynamic efficiency (EFF) that is the response per concentration unit.

### Clinical parameters and safety

Blood pressure measurements and laboratory parameters: blood creatinine, serum symmetric dimethyl arginine (SDMA), urine specific gravity (USG) and urinary protein to creatinine (UPC) ratio were monitored. IRIS staging was determined based on blood creatinine concentration (2, 10). In addition, bodyweight (in kg) and body condition score (BCS) on a 9-point scale (19) were assessed. A 12-point quality of life score (QoL) summed the individual scores (0–3) for attitude, appetite, vomiting and increased drinking and/or urination, a low score indicating a good quality of life.

Finally adverse events were also assessed.

### Statistical analysis for clinical parameters

For weight, laboratory parameters and systolic blood pressure, groups were compared using ANCOVA including baseline as a covariate and country/center as a cofactor.

For BCS, QoL and IRIS stage, groups were compared stratified by country/center using the Cochran-Mantel-Haenszel test.

The 5% level of significance (*p* < 0.05 for two-sided tests) was used to assess statistical differences.

## Results

Table 1 and Figure 1 show that exposure to the computed free benazeprilat (AUCfree) as estimated by the model was significantly lower after administration of the medicated diet than that of Fortekor^®^ and this both in healthy cats (difference between the medians of 52%) and in CKD cats (39%). In a less pronounced way, a lower total benazeprilat exposure (AUCmeasurable) in the MD group was also observed (−26 and −15% respectively).

**Table 1:** Comparison of exposure and efficacy of Fortekor^®^ vs. the medicated benazepril diet based on PK/PD modeling (Bayesian estimates).

Variables (units)	Healthy or CKD cats	Group	Mean	SD	CV%	Min	Median	Max
AUC free (ng^*^h/mL)	Healthy	Fortekor^®^	2,224	892	40.12	989	2,090	3,677
		Medicated diet	1,102	664	60.28	248	1,005	2,227
		**Difference (%)**	**−50.46**				**−51.93**	
	CKD	Fortekor^®^	5,354	6,471	120.85	70	2,999	36,781
		Medicated diet	2,423	2,401	99.10	75	1,816	17,720
		**Difference (%)**	**−54.75**				**−39.45**	
AUC total (ng^*^h/mL)	Healthy	Fortekor^®^	3,543	1,149	32.43	1,693	4,053	4,858
		Medicated diet	3,670	1,558	42.46	2,130	2,991	7,133
		**Difference (%)**	**3.60**				**−26.19**	
	CKD	Fortekor^®^	9,842	6,699	68.06	420	7,426	38,247
		Medicated diet	7,140	4,127	57.80	514	6,309	21,143
		**Difference (%)**	**−27.45**				**−15.05**	
AUEC (U^*^h/mL) *A lower AUEC indicates a higher efficacy (inhibitory effect) on ACE*	Healthy	Fortekor^®^	5,791	2,075	35.84	2,570	6,173	9,374
		Medicated diet	4,490	2,655	59.12	1,110	3,771	9,117
		**Difference (%)**	**−22.46**				**−38.91**	
	CKD	Fortekor^®^	5,472	3,518	64.30	1,110	5,315	27,652
		Medicated diet	5,679	3,600	63.39	1,051	4,946	18,905
		**Difference (%)**	**3.79**				**−6.96**	
Efficiency	Healthy	Fortekor^®^	3.40	2.59	76.21	0.72	2.91	9.48
		Medicated diet	9.75	12.79	131.21	0.50	3.75	36.82
		**Difference (%)**	**186**				**29**	
	CKD	Fortekor^®^	6.23	39.93	640.51	0.07	2.12	396.93
		Medicated diet	9.84	26.95	273.89	0.09	2.61	251.26
		**Difference (%)**	**58**				**23**	

AUC: area under the curve; AUC free is the AUC of free plasma benazeprilat computed by the model and reflects the actual active benazeprilat available in the bloodstream (free benazeprilat exposure); AUC total is the AUC of plasma concentration that is actually measured by the analytical technique and includes both free and bound benazeprilat (total benazeprilat exposure). AUEC is AUC of ACE inhibition measured in inhibitory Unit (U); a lower AUEC indicates a higher efficacy (inhibitory effect) on ACE. Efficiency was measured as the ratio of individual Area under Inhibitory curve for each cat to the corresponding AUC free; higher values mean more effect per amount of benazeprilat.

**Figure 1:**
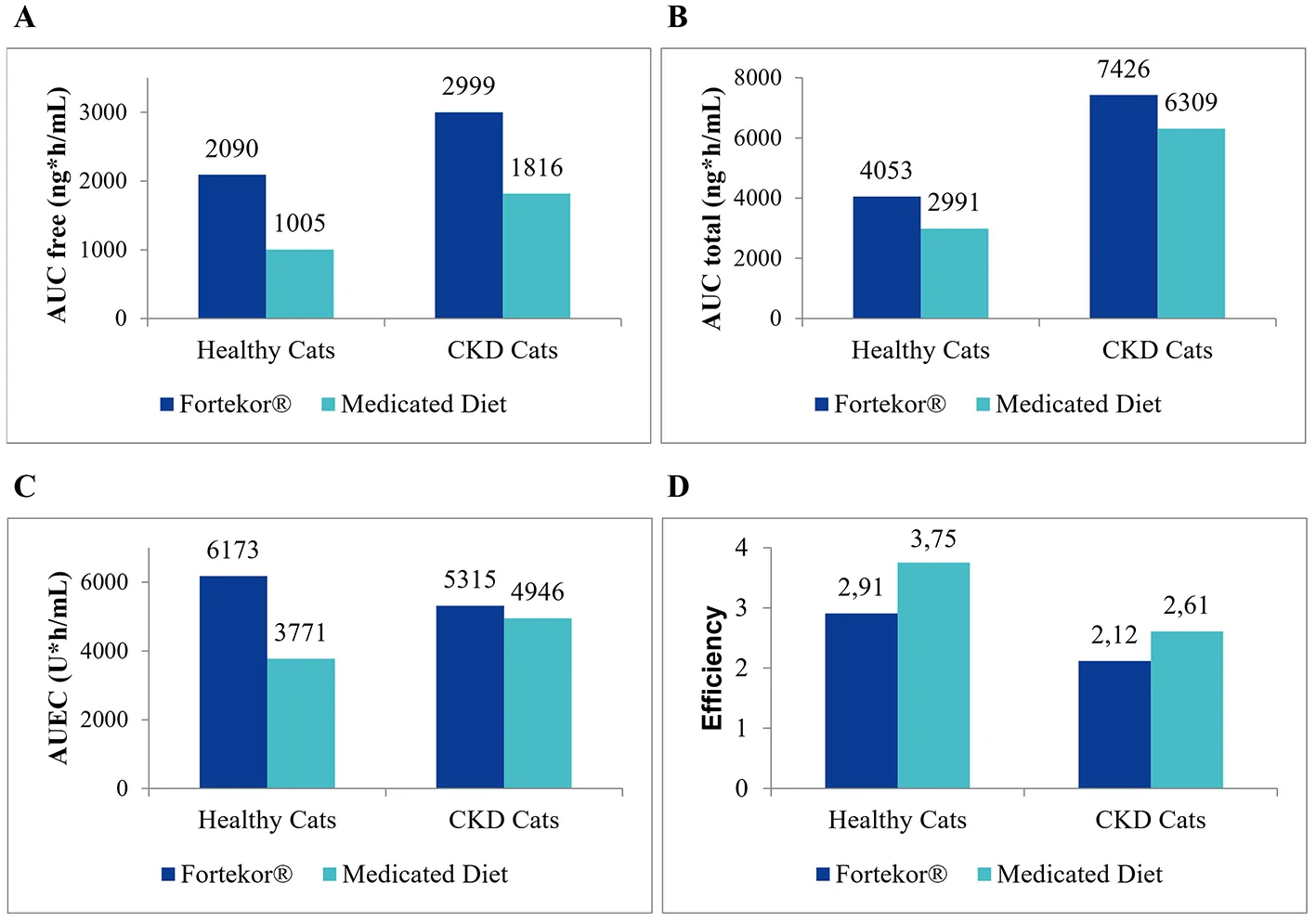
Graphical representation of median exposure and efficacy values of Fortekor^®^ vs. the medicated benazepril diet based on PK/PD modeling: **(A)** Free benazeprilat exposure (AUC free; AUC: Area under the curve) that reflects the actual active benazeprilat available in the bloodstream, **(B)** Total benazeprilat exposure (AUC total) that includes both free and bound benazeprilat, **(C)** ACE inhibition effect (AUEC) that measures efficacy in terms of ACE inhibition, **(D)** Pharmacodynamic efficiency (effect per unit exposure). A lower AUEC indicates a higher efficacy. Higher efficiency means more effect per amount of benazeprilat.

Despite this inferiority in terms of plasma exposure, the inhibitory effects of benazeprilat obtained on ACE activity (AUEC) were more pronounced after the administration of the medicated diet than after administration of Fortekor^®^ in healthy cats (difference between the medians of 39%) and equivalent in CKD cats (7%). As a result, the efficiency of benazeprilat was better in the MD group both in healthy cats (29%) and in CKD cats (23%).

All the clinical parameters, including UPC, remained stable throughout the study and none of them were statistically different between the two groups at the end of the study.

Overall, both products were well tolerated and adverse effects were observed with similar frequency in both treatment groups, mostly signs of deterioration of CKD (anorexia, lethargy) and gastrointestinal disorders (emesis, diarrhea).

## Discussion

This study shows that despite lower exposure to benazeprilat (AUC free) in CKD cats fed the medicated diet, mainly due to a lower ingested dose, the efficacy in terms of ACE inhibition (AUEC) is comparable to the benazepril tablet due to a better efficiency of benazeprilat after its incorporation in a medicated diet. The lower exposure to free benazeprilat in the MD group is explained by an ingested dose of benazepril that can be lower than the nominal dose because it relies on the spontaneous feeding behavior of the cat which can vary from occasion-to-occasion. On the other hand, the entire nominal daily dose of Fortekor^®^ is effectively provided by a forced oral administration imposed by the owner. Advanced stages (especially Stage 3) are associated with fluctuating appetite and altered feeding behavior. IRIS stage alters the pharmacokinetic profile of benazeprilat (decreased clearance for high IRIS stages), but this alteration actually compensates for the lower initial exposure to benazeprilat when the medicated diet is consumed compared to the administration of a tablet. Therefore, the medicated diet can be administered to cats regardless of their IRIS stage and without the need to adjust the treatment. In addition, results from previous preclinical studies showed similar ACE inhibition between cats treated with a full dose of Fortekor and cats eating only 50% of their ration of medicated diet. Therefore, some variability in consumption is possible without impacting ACE inhibition efficacy, provided that at least 50% of the ration is consumed. However, consuming less than 50% of the ration could lead to a lack of efficacy.

To contextualize these findings, it is necessary to understand that drug efficiency does not have the same meaning as drug efficacy. Drug efficiency is defined as the pharmacological effect per unit of drug concentration [See (20) for a review]. When searching to optimize a drug's efficiency, the goal is either to obtain the desired effects with the minimal amount of drug or conversely, for a fixed amount of drug, to obtain an overall maximum cumulative effect. Such a search of the best efficiency is typically addressed using a Pharmacokinetic/Pharmacodynamic (PK/PD) approach and the ultimate goal in establishing PK/PD relationships is precisely to be able to design an optimal dosage regimen which maximizes the effect elicited by a drug per unit dose (i.e., efficiency) (21).

The superior efficiency of the medicated diet is explained by the fact that benazeprilat disposition is strongly nonlinear, that is, there is a non-proportionality between measured plasma concentrations and the administered doses, particularly during the terminal phase when plasma concentrations are low. This is due to the specific and saturable binding to ACE itself. In contrast, when plasma concentrations are above some critical value corresponding to the ACE saturation, the pharmacokinetics of benazeprilat becomes linear and benazeprilat is very quickly eliminated, that is, useless for any pharmacological effect. So the benazeprilat efficiency is highly influenced by its plasma profile and by its administration pattern. When benazepril is administered all at once *via* a tablet, there is a peak plasma concentration in benazeprilat that quickly falls due to elimination, whereas when benazepril is administered through the medicated diet, the rise in plasma concentrations is more gradual giving a dome-shaped plasma concentration profile lasting several hours.

The results confirm what has already been demonstrated by several authors in both dogs and cats, namely that repeated administration of benazepril during the day (every 12 h or even more frequently) rather than a single daily administration is more efficient (15–17).

Over the 180-day period, all the parameters remained stable in the medicated diet group and not different from the control group. This was expected because the cats had already been treated with benazepril for a month, and this confirmed the maintenance of clinical status despite the switch from tablets to the medicated diet. More specifically, the UPC ratio remained unchanged, demonstrating that proteinuria control was maintained with the medicated diet.

Adverse effects were rare in both treatments as expected with benazepril which is a molecule that is very well tolerated in the long term (22). Only a few cats were withdrawn from the study due to side effects: in the MD group in particular, one cat died, three cats showed CKD signs worsening, another developed lipidosis and the last one developed hypertension. No cases of hypotension were reported, which was expected because the hypotensive effect of benazepril is low (23). Furthermore, because the overall free drug exposure is significantly lower with the medicated diet, the risk of concentration-dependent adverse effects, such as hypotension, is naturally minimized.

The classical approach to obtain a slow release profile is to develop an *ad hoc* slow release oral formulation; however tablet formulations are not convenient to administrate on a daily base for very prolonged period of time as required for ACE inhibitor treatment and such a formulation will ineluctably lead to compliance difficulties (11, 12). A new concept was therefore developed incorporating benazepril in a proprietary food vehicle. It was assumed that the fragmentation of daily food intake into several small meals linked to the cat's snacking behavior and associated to the well-established slow gastric emptying in cat of dry food (about 14–16 h) (24) will ensure a plasma concentration profile that avoids the initial peak of plasma concentrations following administration of a tablet that in terms of effects represents lost amounts of benazeprilat; rather we attempted to approximate an ideal smooth oral benazeprilat oral infusion profile. Ultimately, by including benazepril in food, two goals could be achieved simultaneously: increase the pharmacodynamic benazeprilat efficiency in terms of ACE inhibition by smoothing the pharmacokinetic profile of benazeprilat and improve the convenience and compliance of treatment regimen.

This study opens up attractive perspectives regarding the incorporation of medication into food, both in terms of ease of administration and therefore medication compliance and the potential for efficiency gains, depending on the pharmacokinetic behavior of the drug. In the specific case of CKD, for which nutritional management has a major impact on disease progression and life expectancy (2), this allows for the combination of two major pillars of disease management: diet and antiproteinuric treatment. However, the benefits of this new treatment method, including the gain in terms of compliance, can only be obtained if the diet is well accepted and consumed in sufficient quantity; this requires a good dietary transition as well as very good palatability.

## Data Availability

The original contributions presented in the study are included in the article/supplementary material, further inquiries can be directed to the corresponding author.
